# Kinetics and Mechanism of Nanoparticles-Catalyzed Piperidinolysis of Anionic Phenyl Salicylate

**DOI:** 10.1155/2014/604139

**Published:** 2014-11-13

**Authors:** Norazizah Abd. Razak, M. Niyaz Khan

**Affiliations:** Department of Chemistry, Faculty of Science, University of Malaya, 50603 Kuala Lumpur, Malaysia

## Abstract

The values of the relative counterion (*X*) binding constant *R*_*X*_^Br^ (=*K*_*X*_/*K*_Br_, where *K*_*X*_ and *K*_Br_ represent cetyltrimethylammonium bromide, CTABr, micellar binding constants of *X* ^*v*−^ (in non-spherical micelles), *v* = 1,2, and Br^−^ (in spherical micelles)) are 58, 68, 127, and 125 for *X* ^*v*−^ = 1^−^, 1^2−^, 2^−^, and 2^2−^, respectively. The values of 15 mM CTABr/[Na_*v*_ *X*] nanoparticles-catalyzed apparent second-order rate constants for piperidinolysis of ionized phenyl salicylate at 35°C are 0.417, 0.488, 0.926, and 0.891 M^−1^ s^−1^ for Na_*v*_ *X* = Na**1**, Na_2_ **1**, Na**2**, and Na_2_ **2**, respectively. Almost entire catalytic effect of nanoparticles catalyst is due to the ability of nonreactive counterions, *X* ^*v*−^, to expel reactive counterions, **3** ^−^, from nanoparticles to the bulk water phase.

## 1. Introduction

Research on nanoparticles has now become a cutting-edge area of chemical research [1]. Mono- and bilayer surfactant aggregates are nanoparticles which have been known for their characteristic physicochemical properties for more than 100 years [2]. The effects of surfactant aggregates/nanoparticles of different structural features on reaction rates have been extensively studied for the past nearly six decades [3–5]. These studies reveal very complex mechanistic aspects of micellar/nanoparticles catalysis of reaction rates [4–6]. Effects of counterionic salts on ionic surfactant as well as biomolecular structural transitions have been under extensive study since 1887 when Hofmeister first reported specific salt effects on the salting-out proteins [7]. But the mechanistic aspects of these specific salt effects are not yet fully understood [8–10].

Effects of inert salts of moderately hydrophobic counterions, such as benzoate and substituted benzoate ions, on ionic surfactant micellar growth have become very important for various industrial applications [9–11]. However, mechanistic details of such inert salt effects on ionic micellar growth are almost nonexistent. Effects of inert counterionic salts on pseudo-first-order rate constants (*k*_obs_) for the ionic surfactant nanoparticle-catalyzed semi-ionic bimolecular reactions, where ionic reactant is also a counterion, have been explained quantitatively by the use of pseudophase ion-exchange (PIE) model. But the use of PIE model involves mostly counterionic salts of highly and moderately hydrophilic counterions [12]. However, some inherent weaknesses of PIE model have been also realized [13, 14]. The increase in [MX] (MX = 3- and 4-FBzNa with Bz^−^ = C_6_H_4_CO_2_^−^) has caused nonlinear increase in *k*_obs_ for piperidinolysis of anionic phenyl salicylate (PSa^−^) at a constant [CTABr]_*T*_  ≫ cmc where [CTABr]_*T*_ and cmc represent total concentration of cetyltrimethylammonium bromide and critical micelle concentration of CTABr, respectively [15]. The magnitudes of the gradient of the plot of *k*_obs_ versus [MX] show continuous decrease with increasing [MX] [15]. The values of *k*_obs_ remained almost independent of [MX] within its range where the presence of 5 mM CTABr resulted in more than 10-fold increase in *k*_obs_. Thus, 5 mM CTABr/[MX] nanoparticles act as catalyst because, in the absence of CTABr, the values of *k*_obs_ remained independent of [MX] within its range covered in the study [15]. More than 10-fold catalytic effects of CTABr/MX nanoparticles were not emphasized and discussed in the report [15]. The catalytic effects of CTABr/MX/H_2_O nanoparticles catalyst (MX = 4-methoxy and 4-methyl salicylates) on *k*_obs_ for piperidinolysis of PSa^−^ have been studied in the present study. The results and their probable explanations are described in this paper.

## 2. Materials and Methods

### 2.1. Materials

**Figure 1:**
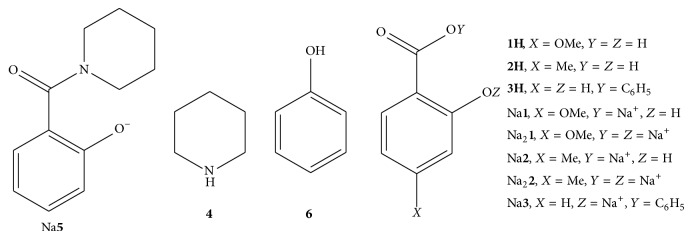
Molecular structures of compounds** 1H**, Na**1**, Na_2_ **1**,** 2H**, Na**2**, Na_2_ **2**,** 3H**, Na**3**,** 4**, Na**5**, and** 6**.

Reagent-grade 4-methoxysalicylic acid (**1H**), 4-methylsalicylic acid (**2H**), cetyltrimethylammonium bromide (CTABr), phenyl salicylate (**3H**), and piperidine (**4**) (Figure 1) were commercial products of highest available purity. Other common chemicals used were also of reagent grade. The stock solutions of 0.50 M M_*v*_ *X* (=Na_*v*_ **1** and Na_*v*_ **2** with *v* = 1 and 2) were prepared by adding 0.52 and 1.25 M NaOH to the corresponding 0.50 M solutions of** 1H** or** 2H**. The stock solutions of 0.01 M** 3H** were prepared in acetonitrile. Throughout the text, the symbol [*X*]_*T*_ represents the total concentration of *X*.

### 2.2. Kinetic Measurements

The rate of CTABr/Na_*v*_ *X* nanoparticles-catalyzed nucleophilic substitution reaction of** 4** with Na**3** was studied spectrophotometrically at 35°C by monitoring the disappearance of Na**3** at 365 or 370 nm. The products of the reaction of** 4** with Na**3** are sodium* N*-piperidinyl salicylate (Na**5**) and phenol (**6**) (Figure 1). The details of the kinetic procedure and product characterization have been described elsewhere [16]. Absorbance values (*A*_ob_) at different reaction time (*t*) were found to fit to (1) for ~8 half-lives of the reactions. In (1), [*R*_0_] represents the initial concentration of** 3H**, *δ*_ap_ is the apparent molar absorptivity of

(1) $$\begin{array}{c} A_{\text{ob}}=\left[R_{0}\right]\delta_{\text{ap}}\exp\left(-k_{\text{obs}}t\right)+A_{\infty } \end{array}$$

the mixture, *k*_obs_ is the pseudo-first-order rate constant, and *A*_*∞*_ = *A*_obs_ at *t* = *∞*. Throughout the study, the initial concentrations of** 3H** or Na**3** were kept constant at 0.2 mM. The choice of this specific concentration was governed by the need to keep it sufficiently low so that it is less than the other salicylate counterions but high enough to measure the absorption spectrophotometrically.

## 3. Results

### 3.1. Effects of [Na_*v*_ *X*] (*v* = 1,2) on *k*_obs_ for the Reaction of **4** with Na**3** at a Constant [CTABr]_*T*_ and 35°C

**Figure 2:**
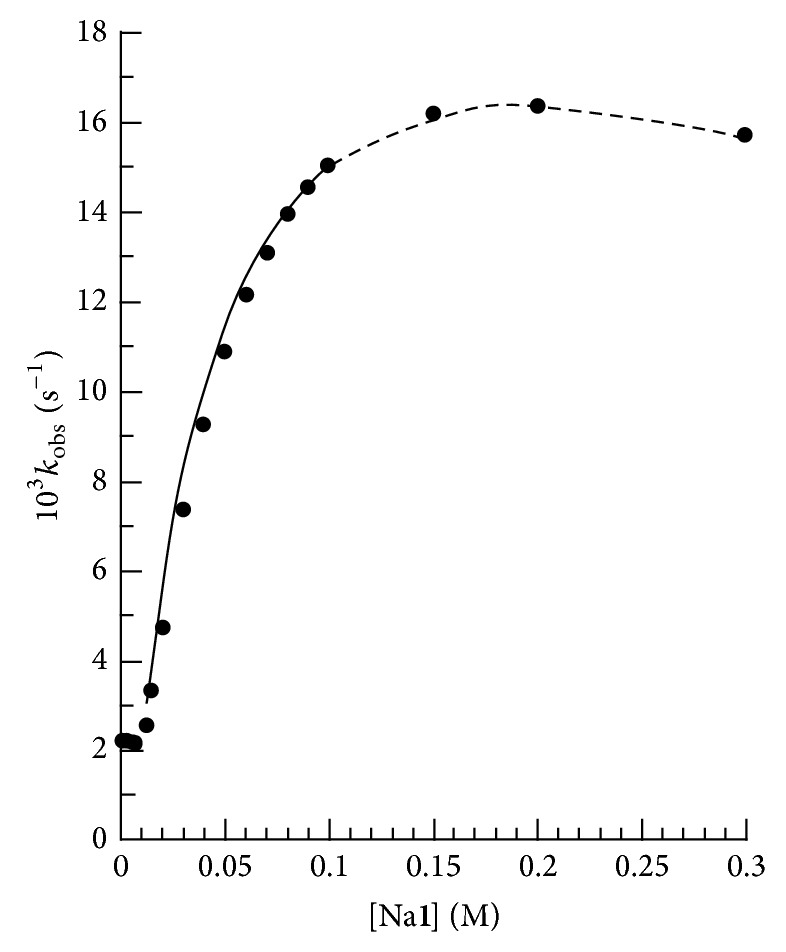
Plots showing the dependence of *k*_obs_ upon [Na**1**] for piperidinolysis of** 3H** at 0.2** **mM** 3H**, 0.1 M** 4**, 0.03 M NaOH, and 35°C. The solid line is drawn through the calculated data points using (2) with kinetic parameters (*k*_cat_ and *K* ^*X*/*S*^), listed in Table 2. The dotted line is drawn through the predicted data points assuming the presence of WM at [Na1]_0_^op^< [Na**1**] ≤ 300 mM.

A series of kinetic runs was carried out at the constant 15 mM CTABr, 0.2 mM** 3H**, 0.1 M** 4**, and varying values of [Na_*v*_ *X*] (*v* = 1,2) within the range 0 ≤ [Na_*v*_ *X*] ≤ 0.30 M for Na_*v*_ *X* = Na_*v*_ **1** (*v* = 1,2). The values of *k*_obs_ versus [Na_*v*_ **1**] at [NaOH]/[**1H**] = 1.04 are shown in Figure 2. Similar plot of *k*_obs_ versus [Na_*v*_ **1**] was also obtained at [NaOH]/[Na_*v*_ **1**] = 2.50. The plot of Figure 2 shows initial segment where the values of *k*_obs_ are almost independent of [Na_*v*_ **1**] at the initial low values of [Na_*v*_ **1**] followed by the segment where the values of *k*_obs_ reveal monotonic increase of more than 7-fold with the increase in [Na_*v*_ **1**].

**Figure 3:**
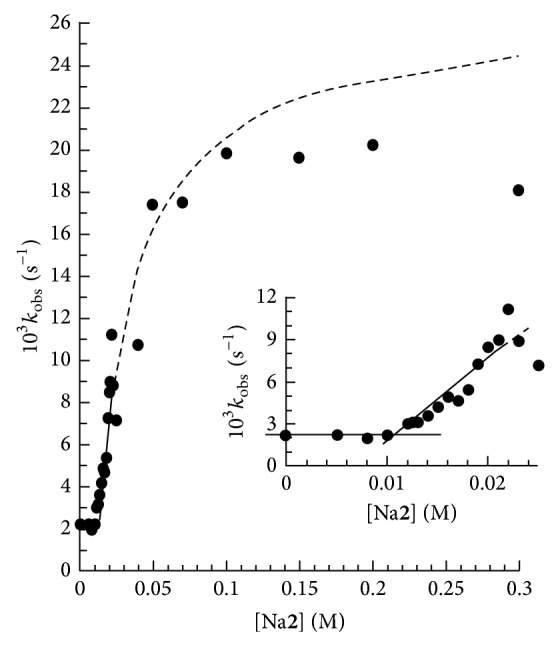
Plot showing the dependence of *k*_obs_ upon [Na**2**], for piperidinolysis of** 3H** at 0.2 mM PSa^−^, 0.1 M** 4**, 0.03 M NaOH, and 35°C. The solid line is drawn through the calculated data points using (2) with kinetic parameters (*k*_cat_ and *K* ^*X*/*S*^), listed in Table 2. The dotted line is drawn through the predicted data points assuming the presence of WM at [Na2]_0_^op^< [Na**2**] ≤ 300 mM.

The values of *k*_obs_ were also obtained at constant 15 mM CTABr, 35°C, 0.2 mM** 3H**, 0.1 M** 4**, and different values of [Na_*v*_ **2**] (*v* = 1,2) within the range 0 ≤ [Na_*v*_ **2**] ≤ 0.30 M. The values of *k*_obs_ versus [Na_*v*_ **2**], at [NaOH]/[**2H**] = 1.04, are shown in Figure 3. Similar plot of *k*_obs_ versus [Na_*v*_ **2**] (not shown) was also obtained at [NaOH]/[**2H**] = 2.5. The values of [NaOH] were varied from 0.030 to ≤0.18 M under the experimental conditions of entire kinetic runs for both Na_*v*_ **1** and Na_*v*_ **2**. The shape of the plot of Figure 3 is similar to that of Figure 2 when [Na_*v*_ **2**] ≤  ~20 mM. The increase in [Na_*v*_ **2**] at ~20 mM Na_*v*_ **2** reveals a mild increase followed by a decrease and then increase again in the values of *k*_obs_ (Figure 3). Similar break in the plot (not shown) of *k*_obs_ versus [Na_*v*_ **2**] was also obtained at [NaOH]/[**2H**] = 2.5. These observations may be attributed to the change in the structure of Na_*v*_ *X*/CTABr nanoparticles to some higher interfacial curvature structures such as curved bilayer structures at ~20 mM Na_*v*_ **2** [17].

The absence and presence of break in the monotonic plot of respective Figures 2 and 3 are indirectly supported by the following observations. The values of *δ*_ap_, obtained for piperidinolysis of** 3** ^−^ at 10 mM NaOH, 100 mM Pip, 0.2 mM** 3H**, 35°C, and 370 nm, increase nonlinearly from 1750 to 4350 M^−1^ cm^−1^ with the increase in CH_3_CN content from 2 to 92% v/v in mixed aqueous solvent (Table 1). The values of *δ*_ap_, obtained for piperidinolysis of** 3** ^−^ at 30 mM NaOH, 100 mM Pip, 0.2 mM** 3H**, 35°C, 370 nm, and different values of [Na_*v*_ *X*], for Na_*v*_ **1** and Na_*v*_ **2** (*v* = 1,2), are also summarized in Table 1. It is evident from Table 1 that (a) the values of *δ*_ap_ are almost independent of [Na_*v*_ *X*] within its range 0–~15 mM for *X* = 1^*v*−^ and 0–~50 mM for *X* = 2^*v*−^ and (b) the values of *δ*_ap_ reveal a monotonic decrease with increasing [Na_*v*_ **1**], *v* = 1,2, within its range ~30–300 mM. But the values of *δ*_ap_ show a sharp decrease with the increase in [Na_*v*_ **2**], *v* = 1,2, from 50 to 70 mM and then become almost independent of [Na_*v*_ **2**] within its range ~70–300 mM. These observations simply demonstrate that Na_*v*_ *X*-induced CTABr/Na_*v*_ *X* nanoparticles structural transition, within [Na_*v*_ *X*] range of 50–300 mM, is not the same for Na_*v*_ **1** and Na_*v*_ **2** (*v* = 1,2).

### 3.2. Effects of [Na_*v*_ *X*] on *k*_obs_ for the Reaction of **4** with Na**3** in the Absence of CTABr at 35°C

In order to quantify the catalytic effects of CTABr/Na_*v*_ *X* nanoparticles on the rate of piperidinolysis of Na**3**, it is essential to study the effects of [Na_*v*_ *X*] on *k*_obs_ at 35°C and [CTABr]_*T*_ = 0. Although benzoate and substituted benzoate ions are nonreactive towards the nucleophilic cleavage of Na**3**, such inert salts might affect *k*_obs_ through ionic strength effect or specific salt effect. Thus, a series of kinetic runs was carried out at 0.2 mM** 3H**, 0.1 M** 4**, 30 mM NaOH, and varying values of [Na_*v*_ **1**] and [Na_*v*_ **2**]. The values of *k*_obs_ reveal <12% decrease within [Na_*v*_ **1**] or [Na_*v*_ **2**] range of 0–100 mM at [NaOH]/[Na_*v*_ **1**] = 1.04 and 0–150 mM at [NaOH]/[ Na_*v*_ **1**] or [Na_*v*_ **2**] = 2.5.

## 4. Discussion

The experimental data (*k*_obs_ versus [Na_*v*_ *X*]) exhibited by Figures 2 and 3 (at [Na_*v*_ **2**] <  ~21 mM) were found to fit to empirical equation:

(2) $$\begin{array}{c} k_{\text{obs}}=\frac{k_{0}+k_{\text{cat}}\left(\left[\text{N}{\text{a}}_{v}X\right]-{\left[\text{N}{\text{a}}_{v}X\right]}_{0}^{\text{op}}\right)}{1+K^{X/S}\left(\left[\text{N}{\text{a}}_{v}X\right]-{\left[\text{N}{\text{a}}_{v}X\right]}_{0}^{\text{op}}\right)}, \end{array}$$

where *k*_cat_ and *K* ^*X*/*S*^ are empirical constants, *k*_0_ = *k*_obs_ at [Na_*v*_ *X*]−[Na_*v*_ *X*]_0_^op^ = 0, and [Na_*v*_ *X*]_0_^op^ represents the optimum concentration of Na_*v*_ *X* below which the values of *k*_obs_ are independent of [Na_*v*_ *X*]. The empirical constant *k*_cat_ represents 15 mM CTABr/[Na_*v*_ *X*] nanoparticles-catalyzed apparent second-order rate constant for piperidinolysis of Na**3**. The values of [Na_*v*_ *X*]_0_^op^ were calculated using an iterative technique as described elsewhere [15]. These values of [Na_*v*_ *X*]_0_^op^ (Table 2) are comparable with the corresponding values of [Na_*v*_ *X*]_0_^op^ obtained by the graphical technique [5]. As described in detail elsewhere [15, 18], the value of [Na_*v*_ *X*]_0_^op^ represents the optimum value of [Na_*v*_ *X*] required for the occurrence of ion exchange processes *X* ^−^/OH^−^ and *X* ^−^/Br^−^. Equation (2), with replacement of *k*_cat_ by *θK* ^*X*/*S*^ where *θ* is an empirical constant, has been found to explain quantitatively similar observed data (*k*_obs_ versus [Na_*v*_ *X*]), for different Na_*v*_ *X* [5]. The nonlinear least-squares technique was used to calculate *k*_cat_ and *K* ^*X*/*S*^ from (2) by considering *k*_0_ as a known parameter. The least-squares calculated values of *k*_cat_ and *K* ^*X*/*S*^ and experimentally determined values of *k*_0_, at [NaOH]/[*X*H] = 1.04 and 2.50, are shown in Table 2. The statistical reliability of the observed data fit to (2) is evident from the standard deviations associated with the calculated values of *k*_cat_ and *K* ^*X*/*S*^ as well as from the solid line plots of Figures 2 and 3 which were drawn through the least-squares calculated data points.

It has been described in detail elsewhere [5, 15, 18] that the nonlinear increase in *k*_obs_ with the increase of [Na_*v*_ *X*] at a constant [CTABr]_*T*_ is due to the transfer of micellized** 3** ^−^ (i.e., 3^−^_*M*_ with subscript *M* indicating micellar pseudophase) to aqueous phase (i.e., 3^−^_*W*_ with subscript *W* indicating bulk water phase) through the occurrence of ion exchange process *X* ^*v*−^/**3** ^−^. This is due to the reason that the value of *k*_obs_ is more than 10-fold larger in the bulk water phase than that in the micellar pseudophase as evident from the listed values of *k*_*W*_^MX^ and *k*_0_ in Table 2. The occurrence of ion exchange *X* ^*v*−^/**3** ^−^ in the related reaction systems [5] has been found to decrease the CTABr micellar binding constant (*K*_*S*_) of** 3** ^−^ with the increasing [Na_*v*_ *X*] through an empirical relationship:

(3) $$\begin{array}{c} K_{S}=\frac{K_{S}^{0}}{\left(1+K_{X/S}\left[\text{N}{\text{a}}_{v}X\right]\right)}, \end{array}$$

where *K*_*S*_^0^ = *K*_*S*_ at [Na_*v*_ *X*] = 0 and *K*_*X*/*S*_ represents an empirical constant whose magnitude is the measure of the ability of counterion *X* ^*v*−^ to expel another counterion *S* ^−^ from the cationic micellar pseudophase to the bulk aqueous phase through the occurrence of ion exchange process *X* ^*v*−^/*S* ^−^ at the cationic micellar surface. It can be easily shown that the reaction mechanism for nucleophilic reaction of** 4** with** 3** ^−^, expressed in terms of pseudophase micellar (PM) model and (3), can lead to (2) [18] with *k*_cat_ and *K* ^*X*/*S*^ expressed by (4) and (5), respectively. As shown in the following equation, *k*_*W*_^MX^ = *k*_obs_ [Na_*v*_ *X*] *k*_obs_ [Na_*v*_ *X*] *F*_*X*/*S*_ is an

(4) $$\begin{array}{c} k_{\text{cat}}=F_{X/S}k_{W}^{\text{MX}}K^{X/S}, \end{array}$$

*k*_*W*_^MX^ = *k*_obs_ obtained within [Na_*v*_ *X*] range where *k*_obs_ values are independent of [Na_*v*_ *X*] in the absence of CTABr and *F*_*X*/*S*_ is an empirical constant whose magnitude should vary in the range >0.0 to ≤1.0 [18]. The following equation

(5) $$\begin{array}{c} K^{X/S}=\frac{K_{X/S}}{\left(1+K_{S}^{0}{\left[\text{CTABr}\right]}_{T}\right)} \end{array}$$

is valid only under the experimental conditions where [CTABr]_*T*_  − cmc ≈ [CTABr]_*T*_ with cmc representing critical micelle concentration of CTABr. Perhaps, it is worth mentioning that the value of cmc of CTABr, at 0.2 mM** 3** ^−^and [Na_*v*_ *X*] = 0, was kinetically determined as 0.09 mM which became 0.04 mM at 0.1 M NaBr. The value of cmc became ~0 at ≥0.5 M NaBr [19]. These observations demonstrate that the value of cmc is negligible compared with [CTABr]_*T*_ at its value of ≥5 mM.

The value of *F*_*X*/*S*_ measures the fraction of the micellized counterions (3_*M*_^−^) transferred to aqueous phase by the optimum concentration of Na_*v*_ *X* through ion exchange *X* ^*v*−^/**3** ^−^ [18]. The value of *F*_*X*/*S*_ was calculated from (4) by the use of listed values of *k*_cat_, *k*_*W*_^MX^, and *K* ^*X*/*S*^ in Table 2 and these calculated values of *F*_*X*/*S*_ for Na**1**, Na_2_ **1**, Na**2,** and Na_2_ **2** are also listed in Table 2. The value of *K*_*X*/*S*_ was calculated from (5) with the reported value of *K*_*S*_^0^ (=7 × 10^3^ M^−1^ [5, 15]). The calculated values of *K*_*X*/*S*_ for Na_*v*_ *X* with *v* = 1,2 and *X* =** 1**,** 2** are shown in Table 2. It has been concluded elsewhere [5, 18] that the normalized *K*_*X*/*S*_^*n*^ (=*F*_*X*/*S*_ *K*_*X*/*S*_) and *K*_*Y*/*S*_^*n*^ (=*F*_*Y*/*S*_ *K*_*X*/*S*_) values are empirically related to the ratio *K*_*X*_/*K*_*Y*_ through the relationship *R*_*X*_^*Y*^ = *K*_*X*_/*K*_*Y*_ = *K*_*X*/*S*_^*n*^/*K*_*Y*/*S*_^*n*^ where *K*_*X*_ = [*X*_*M*_]/([*X*_*W*_][*D*_*n*_]) and *K*_*Y*_ = [*Y*_*M*_]/([*Y*_*W*_][*D*_*n*_]). The symbols *K*_*X*_ and *K*_*Y*_ represent CTABr micellar binding constants of counterions *X* ^−^ and *Y* ^−^, respectively, and [*D*_*n*_] is the concentration of CTABr micelles with each micelle containing *n* number of monomers. The values of *K*_*X*/*S*_^*n*^ (Table 2) and the reported value of 25 M^−1^ [15, 18] for *K*_Br/*S*_^*n*^ (with Br^−^ = Y^−^) give the values of *R*_*X*_^Br^ for *X* = 1^*v*−^, 2^*v*−^ with *v* = 1 and 2. These results are also shown in Table 2. It is relevant to note that the value of *K*_Br/*S*_^*n*^ (=25 M^−1^) is derived from kinetic parameters obtained in the presence of spherical CTABr micelles (SM). But the values of *K*_*X*/*S*_^*n*^ may be derived in the presence of either SM or nonspherical micelles (NSM such as wormlike micelles, WM, or vesicles, Vs). Thus, *R*_*X*_^Br^ becomes conventional ion exchange constant (*K*_*X*_^Br^) if the value of *K*_*X*/*S*_^*n*^ is also obtained in the presence of SM.

The value of *R*_*X*_^Br^ (=68) for *X* = 1^−^ may be compared with the *R*_*X*_^Br^ (=89) obtained at [NaOH]/[*X*H] = 2.1 for *X* representing 5-methoxysalicylate dianion [20]. The reported values of *R*_*X*_^Br^ for *X* = salicylate dianion, benzoate ion, and 4-methoxybenzoate ion are 44, 5.6, and 5.2, respectively [20]. It is evident from the literature that the aqueous solutions of CTABr/*M*_*v*_ *X* containing ≤15 mM CTABr and 12 mM ≤ [*M*_*v*_ *X*] ≤ 22 mM exhibited the presence of SM for *M*_*v*_ *X* = sodium benzoate [21] and WM for *M*_*v*_ *X* = sodium salicylate [22], sodium 3-, 4-, and 5-methyl salicylate [23], and Na_*v*_ **1**, Na_*v*_ **2** where *v* = 1,2. These observations cannot be explained in terms of Hammett substituent constants (σ_H_, *σ*_4-OMe_). These observations reveal that the shapes and sizes of the aqueous CTABr/*M*_*v*_ *X* nanoparticles depend apparently upon the magnitudes of *R*_*X*_^Br^. The magnitude of *R*_*X*_^Br^ is apparently governed by the combined effects of steric requirements and hydrophilic and hydrophobic interactions of counterion *X* ^−^ with cationic headgroup. Hydrophilic interaction includes ion-ion, ion-dipole, dipole-dipole, and inter- and intramolecular hydrogen-bonding interactions.

The values of *k*_cat_ versus *R*_*X*_^Br^ (Table 2) reveal a linear relationship with intercept = 0 and slope = (7.20 ± 0.07) × 10^−3^ M^−1^ s^−1^. This observation implies that almost entire catalytic effect of CTABr/Na_*v*_ *X* nanoparticles catalyst is due to the ability of nonreactive counterions *X* ^*v*−^ to expel the reactive counterions** 3** ^−^ from CTABr/Na_*v*_ *X* nanoparticles to the bulk water phase.

Apparent maximum catalytic constant (*μ*_ap_) of 15 mM CTABr/[Na_*v*_ *X*] nanoparticle catalyst may be obtained from the relationship: *μ*_ap_ = *k*_cat_/*k*_0_ and such calculated values of *μ*_ap_ are 190, (216), 421, and (405 M^−1^) for respective Na**1**, Na_2_ **1**, Na**2**, and Na_2_ **2** where parenthesized values represent at [NaOH]/[*X*H] = 2.5 (i.e., for Na_2_ **1** and Na_2_ **2**). The estimated value of the second-order rate constant (*k*_*M*_^2^) for the reaction of** 4** with** 3** ^−^ in the CTABr micellar pseudophase (i.e., aqueous CTABr nanoparticles), at [Na_*v*_ *X*] = 0, is 3.4 × 10^−3^ M^−1^ s^−1^ [19]. Thus, the real maximum catalytic constants (*μ*_real_) may be obtained from the relationship: *μ*_real_ = *k*_cat_′/*k*_*M*_^2^ where *k*_cat_′ = *k*_cat_/[Pip] (with [Pip] = 0.1 M). The calculated values of *μ*_real_ are 1230, (1440), 2720, and (2620 M^−1^) for respective Na**1**, Na_2_ **1**, Na**2**, and Na_2_ **2** where parenthesized values represent at [NaOH]/[*X*H] = 2.5.

The values of *k*_cat_ and *R*_*X*_^Br^ for Na*X* are not significantly different from the corresponding values for Na_2_ *X* for *X* = 1 and 2 (Table 2). These results reveal that energetically favorable electrostatic interaction is apparently insignificant compared with hydrophobic interaction between counterions, *X* ^*v*−^, and aqueous cationic interface of CTABr/Na_*v*_ *X* nanoparticles. Perhaps, this is the first quantitative explanation of the earlier qualitative experimental observation that sodium salicylate and salicylic acid are equally effective in driving the micellar structural transition SM-to-WM at a constant temperature [23]. The aqueous structure of CTABr/Na_*v*_ *X* nanoparticles remains WM at 35°C, ≤15 mM CTABr and 12 mM ≤ [Na_*v*_ *X*] ≤  ~22 mM for Na_*v*_ *X* = Na_*v*_ **1** and Na_*v*_ **2** (*v* = 1,2). But the values of *k*_cat_ are ~2-fold larger for Na_*v*_ **2** than those for Na_*v*_ **1** (Table 2). Thus, it is apparent that a quantitative correlation between *k*_cat_ and *R*_*X*_^Br^ is better than that between *k*_cat_ and the aqueous structures of CTABr/Na_*v*_ *X* nanoparticles where rheologically assigned structures remain the same (WM) for both Na_*v*_ **1** and Na_*v*_ **2** at <22 mM Na_*v*_ *X*.

## 5. Conclusions

The linear plot of *k*_cat_ versus *R*_*X*_^Br^ with essentially zero intercept reveals indirectly that the catalytic efficiency of CTABr/Na_*v*_ *X*/H_2_O nanoparticles catalyst is almost entirely due to the ability of nonreactive counterions, *X* ^*v*−^, to expel reactive counterions,** 3** ^−^ from nanoparticles to the bulk water phase. Binding affinity of counterions, *X* ^−^ and *X* ^2−^, with CTABr/Na_*v*_ *X*/H_2_O nanoparticles (measured by the magnitude of *R*_*X*_^Br^) remains nearly unchanged for *X* = 1 and 2. The polarity of the CTABr/Na_*v*_ *X* /H_2_O nanoparticles-bound** 3** ^−^ is not the same for *X* ^*v*−^ = 1^*v*−^ and 2^*v*−^, *v* = 1,2, within [Na_*v*_ *X*] range of ~70–300 mM.

## Figures and Tables

**Table 1:** The values of *δ*_ap_, calculated from (1) for the piperidinolysis of 3^−^ under the variety of experimental conditions^a^.

[Na_*v*_ *X*]^b^ (mM)	10^−1^ *δ*_ap_ (M^−1^ cm^−1^)				CH_3_CN (%v/v)	10^−1^ *δ*_ap_ (M^−1^ cm^−1^)^e^
	Na**1** ^c^	Na_2_ **1** ^d^	Na**2** ^c^	Na_2_ **2** ^d^		
0	373 ± 2^f^	369 ± 1^f^	366 ± 2^f^	3372 ± 1^f^	2	175 ± 1^f^
10	380 ± 2	379 ± 1	407 ± 3	386 ± 1	25	215 ± 1
15	356 ± 1	362 ± 1	341 ± 1	343 ± 1	50	250 ± 1
30	323 ± 1	330 ± 1	403 ± 8	395 ± 15	60	265 ± 2
50	286 ± 1	292 ± 2	457 ± 5	364 ± 6	70	288 ± 1
70	276 ± 2	275 ± 2	236 ± 1	250 ± 1	84	300 ± 3
100	251 ± 1	276 ± 1	240 ± 1	230 ± 4	90	367 ± 3
150	239 ± 1	257 ± 1	222 ± 1	227 ± 1	92	435 ± 3
200	230 ± 1	251 ± 1	222 ± 2	226 ± 1		
300	219 ± 1	244 ± 1	221 ± 2	238 ± 2		

^a^[**3H**]_ 0_ = 0.2 mM, *λ* = 370 nm, 35°C, 30 mM NaOH, 100 mM Pip, and 15 mM CTABr. ^b^Na_*v*_ *X* = Na_*v*_1 and Na_*v*_2, *v* = 1, 2. ^c^[NaOH]/[*X*H] = 1.04. ^d^[NaOH]/[*X*H] = 2.50. ^e^Calculated from (1) by the use of observed data (*A*_ob_ versus reaction time *t*) obtained for the kinetic runs at 0.2 mM **3H**, 10 mM NaOH, 100 mM Pip, 370 nm, and 35°C and within CH_3_CN content range of 2–92%v/v in mixed aqueous solvents. ^f^Error limits are standard deviations.

**Table 2:** The values of empirical constants, *k*_cat_ and *K* ^*X/S*^, for Na_*v*_1 and Na_*v*_2 (*v* = 1,2), at 35°C in the presence of CTABr/Na_*v*_ *X* nanoparticles^a^.

Na_*v*_ *X*	[NaOH]/[*X*H]	[Na_*v*_ *X*]_0_^opb^ (mM)	[Na_*v*_ *X*]_0_^opc^ (mM)	10^3^ *k*_*W*_^MX^ ^d^ (s^−1^)	10^3^ *k*_0_^e^ (s^−1^)	10^3^ *k*_cat_ (M^−1^s^−1^)	*K* ^*X/S*^ (M^−1^)	*F*_*X/S*_^f^	*K*_*X/S*_^g^ (M^−1^)	*K*_*X/S*_^*n*^ ^h^ (M^−1^)	*R*_*X*_^Br^ ^i^	[Na_*v*_ *X*]^j^ range (mM)
Na**1**	1.04	11.7	10.6	30.7 ± 0.5^k^	2.20 ± 0.03^k^	417 ± 12^k^	17.7 ± 0.9^k^	0.77	1876	1444	58	12–100
Na_2_ **1**	2.50	13.0	11.6	30.5 ± 0.2	2.26 ± 0.04	488 ± 18	23.5 ± 1.3	0.68	2491	1694	68	15–120
Na**2**	1.04	12.2	10.8	30.3 ± 0.1	2.20 ± 0.03	926 ± 70	30.0 ± 2.6	1.0	3180	3180	127	13–21
Na_2_ **2**	2.50	10.0	9.6	30.3 ± 0.6	2.20 ± 0.03	891 ± 103	30.4 ± 4.2	0.97	3222	3125	125	12–21

^a^[**3H**]_ 0_ = 0.2 mM, *λ* = 365 and 370 nm for Na_*v*_1 and Na_*v*_2, respectively, 30 mM NaOH, 100 mM Pip, and 15 mM CTABr and aqueous reaction mixture for each kinetic run contains 2%v/v CH_3_CN. ^b^Calculated by an iterative technique as mentioned in the text. ^c^Obtained by graphical technique as mentioned in the text. ^d^The value of *k*_*W*_^MX^ is the mean value of *k*_obs_ obtained within [Na_*v*_ *X*] range where *k*_obs_ values remained independent of [Na_*v*_ *X*] at [CTABr]_*T*_ = 0. ^e^The value of *k*_0_ is the mean value of *k*_obs_ values obtained within [Na_*v*_ *X*] range 0.0–≤[Na_*v*_ *X*]_0_^op^ at [CTABr]_*T*_ = 15 mM. ^f^ *F*_*X/S*_ = *k*_cat_/(*k*_*W*_^MX^ × *K* ^*X/S*^). ^g^ *K*_*X/S*_ = *K* ^*X/S*^ × (1 + *K*_*S*_^0^ × [CTABr]_*T*_) where *K*_*S*_^0^ = 7 × 10^3^ M^−1^ and [CTABr]_*T*_ = 15 mM. ^h^ *K*_*X/S*_^*n*^ = *F*_*X/S*_× *K*_*X/S*_. ^i^ *R*_*X*_^Br^ = *K*_*X/S*_^*n*^/*K*_Br/*S*_ with *K*_Br/*S*_ = 25 M^−1^. ^j^Total concentration range of Na_*v*_ *X* used in the data analysis. ^k^Error limits are standard deviations.

## References

[1] Stang P. J. (2012). Abiological self-assembly via coordination: formation of 2D metallacycles and 3D metallacages with well-defined shapes and sizes and their chemistry. *Journal of the American Chemical Society*.

[2] Menger F. M. (1979). The structure of micelles. *Accounts of Chemical Research*.

[3] Fendler J. H., Fendler E. J. (1975). *Catalysis in Micellar and Macromolecular Systems*.

[4] Fendler J. H. (1982). *Membrane Mimetic Chemistry*.

[5] Khan M. N. (2006). *Micellar Catalysis*.

[6] Bunton C. A., Savelli G. (1987). Organic reactivity in aqueous micelles and similar assemblies. *Advances in Physical Organic Chemistry*.

[7] Hofmeister F. (1887). About regularities in the protein precipitating effects of salts and the relation of these effects with physiological behavior of salts. *Archiv für Experimentelle Pathologie und Pharmakologie*.

[8] Parsons D. F., Boström M., Nostro P. L., Ninham B. W. (2011). Hofmeister effects: interplay of hydration, nonelectrostatic potentials, and ion size. *Physical Chemistry Chemical Physics*.

[9] Jungwirth P., Tobias D. J. (2006). Specific ion effects at the air/water interface. *Chemical Reviews*.

[10] Romsted L. S. (2007). Do amphiphile aggregate morphologies and interfacial compositions depend primarily on interfacial hydration and ion-specific interactions? The evidence from chemical trapping. *Langmuir*.

[11] Qi Y., Zakin J. L. (2002). Chemical and rheological characterization of drag-reducing cationic surfactant systems. *Industrial and Engineering Chemistry Research*.

[12] Romsted L. S., Mittal K. L., Lindman B. (1984). Micellar effects on reaction rates and equilibria. *Surfactants in Solutions*.

[13] Germani R., Savelli G., Romeo T., Spreti N., Cerichelli G., Bunton C. A. (1993). Micellar head group size and reactivity in aromatic nucleophilic substitution. *Langmuir*.

[14] Khan M. N., Hubbard A. T. (2002). Mechanism of catalysis in micellar systems. *Encyclopedia of Surface and Colloid Science*.

[15] Yusof N. S. M., Khan M. N. (2010). Determination of an ion exchange constant by the use of a kinetic probe: a new semiempirical kinetic approach involving the effects of 3-F- and 4-F-substituted benzoates on the rate of piperidinolysis of anionic phenyl salicylate in aqueous cationic micelles. *Langmuir*.

[16] Khan M. N., Kun S. Y. (2001). Effects of organic salts on the rate of intramolecular general base-catalyzed piperidinolysis of ionized phenyl salicylate in the presence of cationic micelles. *Journal of the Chemical Society, Perkin Transactions 2*.

[17] Brinchi L., Germani R., Goracci L., Savelli G., Bunton C. A. (2002). Decarboxylation and dephosphorylation in new gemini surfactants. Changes in aggregate structures. *Langmuir*.

[18] Khan M. N. (2010). A new semi-empirical kinetic method for the determination of ion exchange constants for the counterions of cationic micelles. *Advances in Colloid and Interface Science*.

[19] Khan M. N., Arifin Z., Ismail E., Ali S. F. M. (2000). Effects of [NaBr] on the rates of intramolecular general base-catalyzed reactions of ionized phenyl salicylate (PS^-^) with *n*-butylamine and piperidine in the presence of cationic micelies. *The Journal of Organic Chemistry*.

[20] Yusof N. S. M., Khan M. N. (2012). A quantitative correlation of counterion (X) affinity to ionic micelles and X- and temperature-induced micellar growth (spherical-wormlike micelles-vesicles) for X = 5-methyl- and 5-methoxysalicylate ions. *The Journal of Physical Chemistry B*.

[21] Ali A. A., Makhloufi R. (1999). Effect of organic salts on micellar growth and structure studied by rheology. *Colloid and Polymer Science*.

[22] Shikata T., Hirata H., Kotaka T. (1988). Micelle formation of detergent molecules in aqueous media. 2. Role of free salicylate ions on viscoelastic properties of aqueous cetyltrimethylammonium bromide-sodium salicylate solutions. *Langmuir*.

[23] Lin Z., Cai J. J., Scriven L. E., Davis H. T. (1994). Spherical-to-wormlike micelle transition in CTAB solutions. *Journal of Physical Chemistry*.

